# Structural insight into the substrate- and dioxygen-binding manner in the catalytic cycle of rieske nonheme iron oxygenase system, carbazole 1,9a-dioxygenase

**DOI:** 10.1186/1472-6807-12-15

**Published:** 2012-06-24

**Authors:** Yuji Ashikawa, Zui Fujimoto, Yusuke Usami, Kengo Inoue, Haruko Noguchi, Hisakazu Yamane, Hideaki Nojiri

**Affiliations:** 1 Biotechnology Research Center, The University of Tokyo, 1-1-1 Yayoi, Bunkyo-ku, Tokyo, 113-8657, Japan; 2 Protein Research Unit, National Institute of Agrobiological Sciences, 2-1-2 Kannondai, Tsukuba, Ibaraki, 305-8602, Japan; 3 Interdisciplinary Research Organization, University of Miyazaki, 5200 Kihara, Kiyotake, Miyazaki, 889-1692, Japan; 4 Professional Programme for Agricultural Bioinformatics, The University of Tokyo, 1-1-1 Yayoi, Bunkyo-ku, Tokyo, 113-8657, Japan; 5 Education and Research Support Section, Technology Management Division, Administration and Technology Management Center for Science and Engineering, Waseda University, 3-4-1 Okubo, Shinjuku-ku, Tokyo, 169-8555, Japan; 6 Department of Applied Biology and Chemistry, Faculty of Applied Bio-Science, Tokyo University of Agriculture, 1-1-1 Sakuragaoka, Setagaya-ku, Tokyo, 156-8502, Japan

## Abstract

**Background:**

Dihydroxylation of tandemly linked aromatic carbons in a *cis*-configuration, catalyzed by multicomponent oxygenase systems known as Rieske nonheme iron oxygenase systems (ROs), often constitute the initial step of aerobic degradation pathways for various aromatic compounds. Because such RO reactions inherently govern whether downstream degradation processes occur, novel oxygenation mechanisms involving oxygenase components of ROs (RO-Os) is of great interest. Despite substantial progress in structural and physicochemical analyses, no consensus exists on the chemical steps in the catalytic cycles of ROs. Thus, determining whether conformational changes at the active site of RO-O occur by substrate and/or oxygen binding is important. Carbazole 1,9a-dioxygenase (CARDO), a RO member consists of catalytic terminal oxygenase (CARDO-O), ferredoxin (CARDO-F), and ferredoxin reductase. We have succeeded in determining the crystal structures of oxidized CARDO-O, oxidized CARDO-F, and both oxidized and reduced forms of the CARDO-O: CARDO-F binary complex.

**Results:**

In the present study, we determined the crystal structures of the reduced carbazole (CAR)-bound, dioxygen-bound, and both CAR- and dioxygen-bound CARDO-O: CARDO-F binary complex structures at 1.95, 1.85, and 2.00 Å resolution. These structures revealed the conformational changes that occur in the catalytic cycle. Structural comparison between complex structures in each step of the catalytic mechanism provides several implications, such as the order of substrate and dioxygen bindings, the iron-dioxygen species likely being Fe(III)-(hydro)peroxo, and the creation of room for dioxygen binding and the promotion of dioxygen binding in desirable fashion by preceding substrate binding.

**Conclusions:**

The RO catalytic mechanism is proposed as follows: When the Rieske cluster is reduced, substrate binding induces several conformational changes (e.g., movements of the nonheme iron and the ligand residue) that create room for oxygen binding. Dioxygen bound in a side-on fashion onto nonheme iron is activated by reduction to the peroxo state [Fe(III)-(hydro)peroxo]. This state may react directly with the bound substrate, or O–O bond cleavage may occur to generate Fe(V)-oxo-hydroxo species prior to the reaction. After producing a *cis*-dihydrodiol, the product is released by reducing the nonheme iron. This proposed scheme describes the catalytic cycle of ROs and provides important information for a better understanding of the mechanism.

## Background

Many studies have demonstrated that aromatic ring dihydroxylation plays a primary role in the initial step of aerobic bacterial degradation pathways for various natural and synthetic aromatic compounds, including dioxins, polychlorinated biphenyls, and crude oil components such as polycyclic aromatic hydrocarbons and heterocyclic aromatic compounds [1-7]. Such ring dihydroxylation is catalyzed by multicomponent oxygenase systems known as Rieske nonheme iron oxygenase systems (ROs). A member of ROs, called as aromatic ring-hydroxylating dioxygenase, catalyze the incorporation of both oxygen atoms of molecular dioxygen as two hydroxyl groups to tandemly linked carbon atoms of an aromatic ring in a *cis*-configuration. The introduction of both oxygen atoms into aromatic substrates is a key reaction to initiate the transformation of relatively unreactive aromatic compounds, and novel oxygen activation and the addition mechanism of ROs is a subject of great interest. Despite substantial progress in understanding the structure, regulation, and kinetics of ROs in recent studies [8], no consensus exists on the chemical steps in the catalytic cycle. Further knowledge of the mechanism can lead us to improved application of this important class of enzymes for not only the bioremediation of various environmentally relevant aromatic compounds, but also the synthesis of the chiral precursor compounds [9].

The multicomponent systems of ROs typically comprise the terminal oxygenase component and the electron transport chain, which consists of either one or two separate proteins: reductase alone or ferredoxin and reductase in combination [1]. Biochemical and/or structural properties of several terminal oxygenase components of RO (RO-Os) have demonstrated that RO-Os have a mononuclear iron site and a Rieske-type [2Fe-2 S] cluster (Rieske cluster) in common [10]. The mononuclear iron site activates dioxygen for reaction with the substrate, and the Rieske cluster transfers electrons to the mononuclear iron site during the catalytic cycle. The structures of RO-Os reveal that each α-subunit contains a mononuclear iron site and a Rieske cluster separated by a distance of approximately 45 Å. However, the functional pair appears to be constituted by the mononuclear iron and the Rieske cluster in neighboring subunits located within ~12 Å distance. The nonheme iron site is coordinated by two histidine residues and one carboxylate residue called the 2-His-1-carboxylate facial triad, which is a versatile platform of nonheme iron-containing oxygenases including ROs [11]. In most RO-O structures, the carboxylate residue is coordinated to the iron in a bidentate manner [12-17], although monodentate structures have also been reported [18-22]. Additional water molecules are found to be coordinated to the nonheme iron, forming a five- or six-coordinate catalytic ferrous site, depending on the number of water molecules. The Rieske cluster has two iron and two sulfides; one iron is coordinated by two histidine residues, and the other is coordinated by two cysteine residues. The nonheme iron site and the Rieske cluster can be bridged by a conserved aspartate residue located at the subunit interface, which may be important for electron transfer [23] and regulation [24] during the catalytic cycle.

The metal site composition of RO-Os generally suggests that three oxidation states can stably exist: both metal sites are oxidized, the Rieske cluster is oxidized and mononuclear iron is reduced, and two metal sites are reduced. Another potential oxidation state with a reduced Rieske cluster and oxidized mononuclear iron is not stable because of the relative redox potentials of the metal sites. Spectroscopic studies report that substrate binding and dioxygen activation occur at the mononuclear iron center for naphthalene 1,2-dioxygenase (NDO) and benzoate 1,2-dixoygenase (BZDO) [25-28]. Wolf et al. demonstrated that the oxygenase component of BZDO (BZDO-O) alone in the fully reduced state could activate dioxygen and generate the *cis*-dihydrodiol product in a single turnover reaction [26]. Following the single turnover of BZDO-O, this protein was found to be in the fully oxidized state with most of the products retained in the active site, suggesting that the mononuclear iron and Rieske cluster each provide one of two electrons required by the reaction stoichiometry. The same was found to be true for the oxygenase component of NDO (NDO-O), which produced an essentially stoichiometric yield of product in a single turnover based on the number of populated mononuclear irons present [25]. The binding of the dioxygen to the ferrous catalytic site was regulated by both substrate binding and Rieske cluster reduction, implying that a structural change occurs in the vicinity of the ferrous iron site during the catalytic cycle. Indeed, this was demonstrated from crystallography for an allosteric effect of the Rieske cluster in 2-oxoquinoline 8-monooxygenase (OMO) [14]. Martins et al. showed that reduction of the Rieske cluster modulated the ferrous nonheme iron through a chain of structural changes across the subunit interface, resulting in the movement of the nonheme iron and its ligand histidine away from a substrate-binding site [14]. The ferrous nonheme iron changes from five- to six-coordinate, which was also found for NDO [25], BZDO [26], and phthalate 4,5-dioxygenase (PDO) [24,29]. However, the crystal structures of the ferrous nonheme iron in NDO [13] and carbazole (CAR) 1,9a-dioxygenase (CARDO) [16] with a reduced Rieske cluster are five-coordinate, which differs from OMO-O. In addition, binding of the substrate to the active site of RO-Os is a key step in regulating the reactivity toward dioxygen. Until now, spectroscopic studies on NDO-O and the oxygenase component of PDO (PDO-O) found that the NDO-O (Rieske cluster is either oxidized or reduced) and PDO-O with the oxidized Rieske cluster showed conversion of ferrous iron from six- to five-coordinate upon binding of the substrate to the active site [30-32]. However, the crystal structures of the nonheme iron site in the native and substrate-bound forms of NDO-O did not indicate a significant change in the coordination environment, maintaining five-coordinate structures [13]. The five-coordinate nonheme iron in the oxygenase component of OMO (OMO-O) with the oxidized Rieske cluster also did not show a change in coordination number even when a substrate was bound to the active site [14]. On the other hand, Daughtry et al. have reported that five-coordinate geometry leaves one site open for oxygen binding and activation or the single solvent ligand could be displaced by oxygen binding, compared between the unliganded and the substrate binding structures of stachydrine demethylase [33]. As noted above, spectroscopy and current crystal structures have provided different perspectives of the catalytic mechanism involved in ROs.

The observation that two electrons present in the Rieske cluster and the mononuclear iron of NDO-O and BZDO-O are used during a single turnover supports the mechanism by which dioxygen is activated by reduction to the peroxo state after binding to the active site iron [Fe(III)-(hydro)peroxo]. Following dioxygen activation, this state is considered to react directly with bound substrate or to result in O–O bond cleavage generating Fe(V)-oxo-hydroxo species prior to reaction [8,34,35]. An alternative mechanism involves an additional step in which one electron is donated to the initially formed Fe(III)-(hydro)peroxo to produce the Fe(II)-(hydro)peroxo intermediate, which could react either directly or as Fe(IV)-oxo-hydroxo species after O–O bond cleavage. The latter mechanism is supported by studies that used PDO-O in which the mononuclear iron was left in Fe(II) state after yielding product [29,36], while single turnover occurred as observed for NDO-O and BZDO-O. These studies suggest that an additional electron was transferred to the nonheme iron at some stage of the catalytic cycle from a Rieske cluster in a neighboring α-subunit.

To understand the molecular basis of the catalytic cycle, Karlsson et al. formed complex crystals of NDO-O with substrate, dioxygen, substrate plus dioxygen, or product and determined their structures by X-ray crystallography [13]. The complex structure with substrate and dioxygen showed that dioxygen was bound to the nonheme iron in a side-on fashion, which allowed each oxygen atom to attack the neighboring aromatic carbon atom from the same face of a planar aromatic ring, producing a *cis*-dihydrodiol. In addition, based on the Fe–O bond lengths in the crystal structure, the complex is likely to be an Fe(III)-(hydro)peroxo species. However, conformational changes by the binding of substrate and/or oxygen in the above-mentioned crystal structures were hardly observed. In these situations, determining whether conformational changes including coordination conversion at the active site occur by binding of substrate and/or oxygen is very important for a better understanding of the catalytic mechanisms in ROs.

We have investigated the enzymatic function of CARDOs, members of ROs, from various bacteria: *Pseudomonas resinovorans* CA10, *Janthinobacterium* sp. J3, *Novosphingobium* sp. KA1, and *Nocardioides aromaticivorans* IC177 [7,37]. All CARDOs commonly consist of three components: terminal oxygenase (CARDO-O), ferredoxin (CARDO-F), and ferredoxin reductase (CARDO-R). We determined the structures of CARDO-Os of J3 and IC177 [15,17] and CARDO-Fs of CA10 and IC177 [17,38]. In addition, the structures of the CARDO-O: CARDO-F binary complex were determined in non-reduced, reduced, and CAR-bound forms using CARDO-O of J3 and CARDO-F of CA10 [16]. These structures provide a structure-based interpretation of inter-component electron transfer between two Rieske clusters of ferredoxin and oxygenase in ROs as well as, conformational changes upon CAR binding, which result in the closure of a lid over the substrate-binding pocket [16].

In the present study, we used CARDO-O of J3 and CARDO-F of CA10, hereafter simply termed Oxy and Fd, respectively, and aimed to clarify the holistic catalytic mechanism including conformational changes in ROs by determining various structures of the Oxy:Fd binary complex at different steps.

## Results and discussion

### Overview

**Figure 1:**
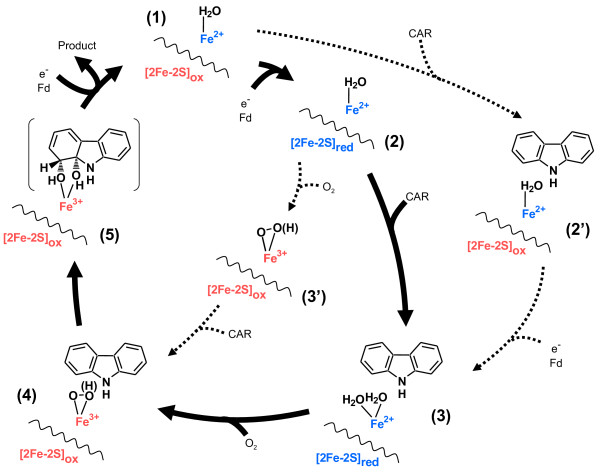
**Proposed catalytic cycle of CARDO on the basis of various Oxy:Fd complex structures.** Statuses are as follows: (1) the resting state with the oxidized Rieske cluster and the ferrous nonheme iron (Oxy: Fd^rest^, PDB code 2DE5) [16]; (2) the reduced state (Oxy: Fd^red^, PDB code 2DE6) [16]; (2’) the CAR-bound state (Oxy: Fd^CAR^, PDB code 2DE7) [16]; (3) the reduced CAR-bound state (Oxy: Fd^red-CAR^, PDB code 3VMG); (3’) the oxygen-bound state (Oxy: Fd^O2^, PDB code 3VMH); (4) the CAR- and oxygen-bound state (Oxy: Fd^CAR-O2^, PDB code 3VMI); and (5) the product-bound state. [2Fe-2 S] shows the coupling Rieske [2Fe-2 S] cluster of the neighboring Oxy subunit, and nonheme iron ligands in the active site are omitted. Solid arrows indicate the proposed main pathway in the catalytic cycle, and broken arrows show the possible pathway based on the complex structures determined in previous and present studies. Red and blue letters show the oxidized and reduced states of redox centers, respectively. The subscripts “ox” and “red” also indicate the oxidized and reduced states, respectively, of the Rieske [2Fe-2 S] cluster.

**Figure 2:**
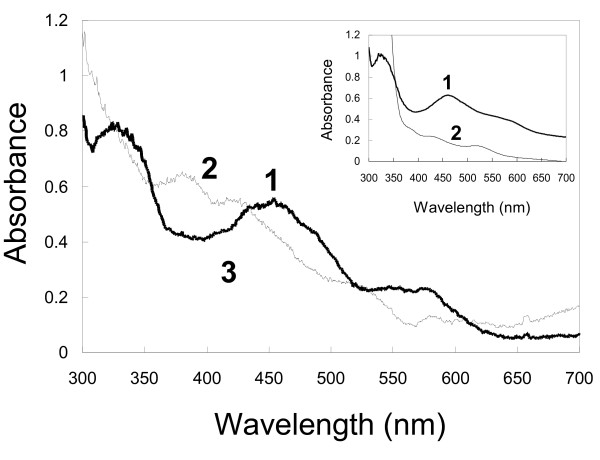
**UV-visible absorption spectra of the Rieske cluster in crystals and the mixed solution.** The absorption spectra of crystals of the resting state [Oxy: Fd^rest^, (1) in Figure 1, reduced state [Oxy: Fd^red^, (2)], and oxygen-bound state [Oxy: Fd^O2^, (3’)] under a cryo-stream of nitrogen at 100 K are shown in curves 1, 2, and 3, respectively, in the main panel. In the inset, curve 1 in the small panel shows the non-reduced spectrum of the mixed solution of purified CARDO-O and CARDO-F, which were used for crystallization. Curve 2 in the inset represents the spectrum after the addition of sodium dithionite to the same mixed solution. (Curves 1 and 2 from both crystal and solution are referred from our previous study) [16].

Although Oxy:Fd binary complexes in non-reduced, reduced, and non-reduced CAR-bound forms were called Binary Complex, Complex^red^, and Complex^subs^, respectively, in a previous paper (PDB code 2DE5, 2DE6, and 2DE7) [16], they were respectively renamed Oxy: Fd^rest^ [(1) in Figure 1, Oxy: Fd^red^ (2), and Oxy: Fd^CAR^ (2’) in this paper, because the non-reduced complex can be considered to be in the resting state in the catalytic cycle and because the CAR-bound complex was obtained using resting state crystals soaked in a CAR-containing crystallization solution under aerobic conditions. UV-visible absorption spectra of the crystals of Oxy: Fd^rest^ and Oxy: Fd^red^ were previously measured by single-crystal microspectrophotometer [39], suggesting that Rieske clusters in Oxy: Fd^rest^ and Oxy: Fd^red^ were oxidized and reduced, respectively (Figure 2) [16]. We did not measure the absorption spectra of crystals soaked with CAR-containing solution. But the redox state of the Rieske cluster in Oxy: Fd^CAR^ was considered to be oxidized, because of the fact that Oxy: Fd^CAR^ was obtained by soaking with CAR-containing solution of resting state crystals at atmospheric condition. On the other hand, this spectroscopic analysis did not provide information on the redox state of the nonheme iron.

In the present study, we determined the crystal structures of the reduced CAR-bound Oxy:Fd complex (Oxy: Fd^red-CAR^), oxygen-bound Oxy:Fd complex (Oxy: Fd^O2^), and both CAR- and oxygen-bound Oxy:Fd complex (Oxy: Fd^CAR-O2^) at 1.95, 1.85, and 2.0 Å resolution, respectively. To ensure redox state of the Rieske cluster of the crystals, absorption spectra of oxygen-bound Oxy:Fd crystal was measured as described previously [16]. An absorption pattern similar to that of Oxy: Fd^rest^ crystal was observed, suggesting that Rieske clusters in oxygen-bound Oxy:Fd (Oxy: Fd^O2^) crystals were re-oxidized by dioxygen binding/exposure (Figure 2). Although we did not determined the absorption spectra of CAR-soaked Oxy:Fd crystals, the Rieske clusters of Oxy: Fd^red-CAR^ and Oxy: Fd^CAR-O2^ crystals were presumed to be reduced and oxidized, respectively, because the methods for dithionite treatment and O_2_ exposure were similar to those in preparation of Oxy: Fd^red^ and Oxy: Fd^O2^, respectively. Statuses around ferrous iron in the active site and the Rieske cluster of the Oxy: Fd^red-CAR^, Oxy: Fd^O2^, and Oxy: Fd^CAR-O2^ are (3), (3’), and (4), respectively, in Figure 1. Crystal data and refinement statistics of the three determined Oxy:Fd structures in this paper are summarized in Table 1. All Oxy:Fd structures solved consist of one molecule of Oxy (comprising three subunits: chains A, B, and C) and three molecules of Fd (chains D, E, and F), and the asymmetric unit of the crystal contains one Oxy:Fd structure. Three molecules of Fd bind to the subunit boundary of one Oxy molecule. The superposition of three Fd molecules binding to one Oxy molecule in each Oxy:Fd structures resulted in a root mean square deviation (RMSD) of 0.47-0.51 Å (104 Cα atoms) and no evident conformational differences. In addition, comparisons of Fd structures among the three structures also revealed no clear conformational changes (RMSD, 0.16-0.19 Å in 104-107 Cα atoms) with the exception of a clear movement of the side chain of Fd Phe67, which was observed in the Oxy: Fd^red^ crystal in response to reduction of crystal previously [16]. In contrast, some recognizable conformational changes were observed resulting from reduction/substrate-binding around the active site iron and in the substrate-binding pocket of Oxy subunits of these Oxy:Fd structures. Structural differences among the three subunits of one Oxy molecule mainly occurred by CAR binding. The electron density maps of CAR were only found in chains B and C, because the symmetry-related structure partially filled the entrance of the substrate-binding pocket in chain A (Table 2).

**Table 1:** Crystal data and refinement statistics of the three Oxy:Fd complex structures

	**Reduced CAR-bound binary complex**	**Oxygen-bound binary complex**	**CAR- and oxygen-bound binary complex**
	**(Oxy: Fd**^**red-CAR**^**)**	**(Oxy: Fd**^**O2**^)	**(Oxy: Fd**^**CAR-O2**^**)**
**Crystal Data**			
Space group	*P*2_1_	*P*2_1_	*P*2_1_
Unit cell parameters (Å, °)	*a* = 98.2, *b* = 89.8,	*a* = 98.2, *b* = 89.4,	*a* = 98.0, *b* = 89.6,
	*c* = 105.2,	*c* = 105.0,	*c* = 104.8,
	β = 104.4	β = 104.1	β = 104.2
Completeness (%)^*a*^	99.9 (99.9)	99.9 (99.4)	99.3 (97.6)
*R*_merge_^*b*^ (%)^*a*^	8.1 (33.8)	6.0 (36.3)	6.3 (31.2)
Resolution range (Å)^*a*^	50.0-1.95 (2.02-1.95)	50.0-1.85 (1.92-1.85)	50.0-2.00 (2.07-2.00)
Total no. of reflections	684,339	841,651	422,706
No. of unique reflections	128,789 (12,781)	150,169 (14,838)	117,108 (11,459)
**Refinement**			
Resolution range (Å)^*a*^	40.6-1.95 (2.02-1.95)	47.6-1.85 (1.92-1.85)	32.7-2.00 (2.07-2.00)
*R*-factor (%)^*c*^	19.8	19.6	20.5
*R*_free_ (%)^*d*^	21.9	22.5	24.0
Root mean square deviation (RMSD)			
Bond length (Å)	0.005	0.005	0.006
Bond angles (°)	1.3	1.3	1.3
Model composition and average B factor (Å^2^)			
All	2521, 29.2	2716, 32.0	2337, 40.6
Residues	1482, 28.6	1483, 31.1	1481, 40.2
Water	1037, 36.3	1230, 40.4	851, 45.6
CAR	2, 28.8	–, –	2, 48.2
Dioxygen	–, –	3, 36.4	3, 50.9
Ramachandran plot^*e*^			
Favored region (%)	96.4	95.9	95.4
Allowed region (%)	3.4	3.9	4.4
Outlier region (%)	0.2	0.2	0.3

^*a*^ Values in parentheses are for the outermost shell.

^*b*^*R*_merge_ = Σ_**h**_ Σ_*l*_ |*I*_**h***l*_ - < *I*_**h**_ > |/Σ_**h**_ Σ_*l*_ < *I*_**h**_*>*, where *I*_*l*_ is the *l*th observation of reflection **h** and < *I*_**h**_ > is the weighted average intensity for all observations *l* of reflection **h**.

^*c*^*R*-factor is defined as *R* = Σ||F_obs_| - |F_calc_||/Σ|F_obs_|.

^*d*^*R*_free_ was calculated using 5% of the unique reflections.

^*e*^ Ramachandran plot was analyzed with RAMPAGE [40].

**Table 2:** Substrate-binding statuses in each subunit of Oxy in the catalytic cycle

**State**^***a***^	**Oxy subunits**		
	**Chain A**	**Chain B**	**Chain C**
(1) Resting (Oxy: Fd^rest^)	-	-	-
(2) Reduced (Oxy: Fd^red^)	-	-	-
(2’) CAR-bound (Oxy: Fd^CAR^)	-	CAR	CAR
(3) Reduced CAR-bound (Oxy: Fd^red-CAR^)	-	CAR	CAR
(3’) Oxygen-bound (Oxy: Fd^O2^)	Peroxide (end-on)	Peroxide (end-on)	Peroxide (end-on)
(4) CAR- and oxygen-bound (Oxy: Fd^CAR-O2^)	Peroxide (end-on)	Peroxide (side-on)	Peroxide (side-on)
		+CAR	+CAR

^*a*^ Numbers correspond to Figure 1.

### The reduced oxy:Fd complex structure bound with CAR [oxy: Fd^red-CAR^, (3)]

**Figure 3:**
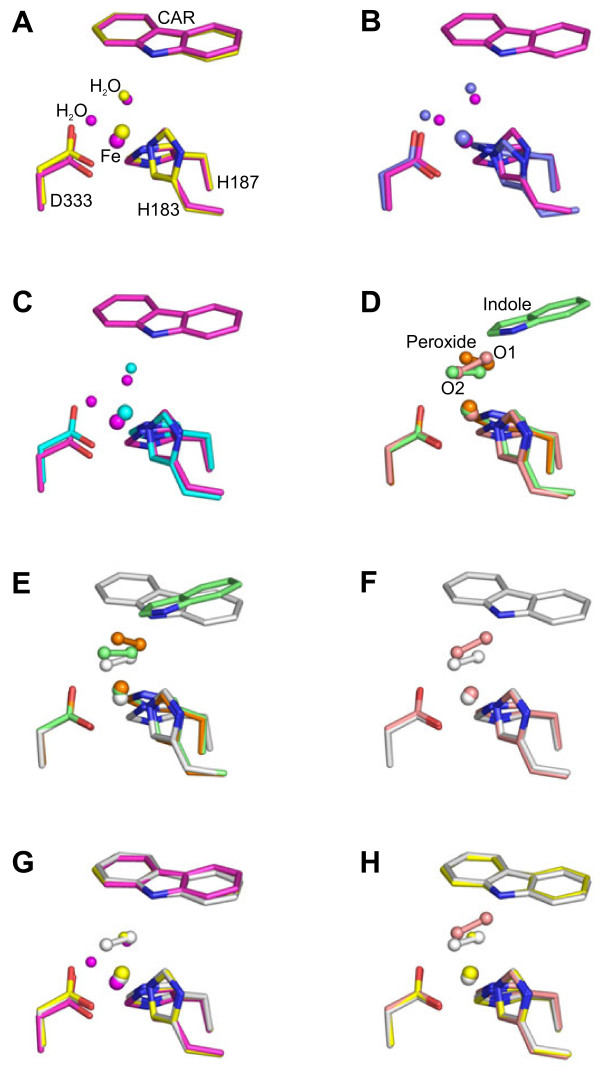
**Superimpositions of the active site of chain C in complex structures.** (**A**) Oxy: Fd^red-CAR^ [(3), magenta] and Oxy: Fd^CAR^ [(2’), yellow]. Only the nonheme iron (sphere), waters liganded to the iron (small sphere), iron-coordinated residues (H183, H187, and D333; stick), and CAR (stick) are shown. (**B**) Oxy: Fd^red-CAR^ [(3), magenta] and OMO-O in the reduced state (PDB code 1Z02, slate) [14]. (**C**) Oxy: Fd^red-CAR^ [(3), magenta] and Oxy: Fd^red^ [(2), cyan]. (**D**) Oxy: Fd^O2^ [(3’), salmon], NDO-O bound with dioxygen only (PDB code 1O7M, orange), and both oxygen and indole (PDB code 1O7N, lime) [13]. Dioxygen molecule and indole are shown as stick-and-ball and stick, respectively. (**E**) Oxy: Fd^CAR-O2^ [(4), white], NDO-O bound with oxygen only (PDB code 1O7M, orange), and both oxygen and indole (PDB code 1O7N, lime) [13]. (**F**) Oxy: Fd^CAR-O2^ [(4), white] and Oxy: Fd^O2^ [(3’), salmon]. (**G**) Oxy: Fd^CAR-O2^ [(4), white], Oxy: Fd^CAR^ [(2’), yellow], and Oxy: Fd^red-CAR^ [(3), magenta]. (H) Oxy: Fd^CAR-O2^ [(4), white], Oxy: Fd^CAR^ [(2’), yellow], and Oxy: Fd^O2^ [(3’), salmon].

Using Oxy: Fd^rest^ crystals soaked in a crystallization solution containing sodium dithionite and CAR, we obtained the structure of reduced Oxy:Fd complex bound with CAR, Oxy: Fd^red-CAR^ [(3) in Figure 1. Oxy: Fd^red-CAR^ provided the same observations as those of Oxy: Fd^CAR^ (2’), which were a clear density for a flat aromatic compound in the active site (Additional file 1: Figure S1A and Table 2) and the CAR-binding-dependent movement of amino acid residues Leu202–Thr214 and Asp229–Val238 toward the entrance of the substrate-binding pocket in chains B and C (data not shown) [16]. A comparison of the structures between Oxy: Fd^red-CAR^ (3) and Oxy: Fd^rest^ (1)/Oxy: Fd^CAR^ (2’) revealed a shift of approximately 0.7 Å in the phenyl group of Fd Phe67 opposite the Rieske cluster. The slight movement of Phe67 in Oxy: Fd^red-CAR^ was common in Oxy: Fd^red^, in which the single-crystal microspectrophotometer clearly confirmed the reduced status of the Rieske cluster [16]. This suggests that this slight movement of Phe67 may be specific for reduction of the Rieske cluster. The active site of Oxy: Fd^red-CAR^ showed a six-coordinate, distorted octahedral geometry with two water ligands, while that of Oxy: Fd^CAR^ (2’) could be described as a distorted tetragonal geometry with one water ligand (Figure 3A). Although one water ligand was located at almost the same position as the water ligands of the Oxy: Fd^CAR^ structure, the location of the other had moved toward Asp333, the ligand of the nonheme iron, and the observed average bond length between two water molecules was 2.7 Å. The position of the two water ligands was similar to that in the reduced OMO-O structure obtained by crystallization under anoxic conditions (Figure 3B).

**Figure 4:**
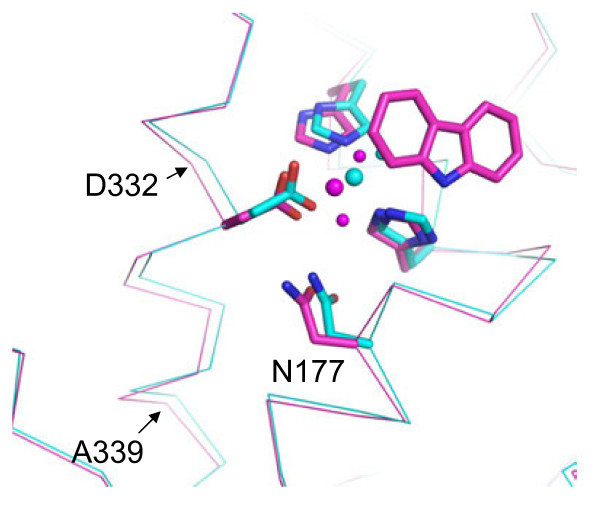
**Conformational changes around the nonheme iron observed between reduced two Oxy:Fd structures.** Superposition of Oxy: Fd^red-CAR^ [(3) in Figure 1, magenta] and Oxy: Fd^red^ [(2), cyan] when CAR is bound to the active site with the reduced Rieske cluster is shown as in Figure 3.

Orientations of CAR were virtually identical between Oxy: Fd^red-CAR^ and Oxy: Fd^CAR^ as shown in Figure 3A, clearer density and lower temperature factors were observed under the reduced condition (substrate’s average temperature factors: 28.8 Å^2^ in reduced crystal and 35.1 Å^2^ in non-reduced crystal), suggesting that CAR is bound to the active site more stably when the Rieske cluster is reduced. Notably, the nonheme iron and its ligand residue, Asp333, moved away from CAR by approximately 0.5 and 0.4 Å, respectively, in Oxy: Fd^red-CAR^ (Figure 3A). In addition, compared with Oxy: Fd^red^ (2), the movements of the nonheme iron and Asp333 upon CAR binding were approximately 0.9 and 0.7 Å, respectively (Figure 3C). Small conformational changes were also observed in residues around Asp333 (residues 332–339) on the distorted helix α11, forming the active site, with shifts between 0.3 and 0.8 Å for the Cα atoms (Figure 4). The histidine ligands adapted to the movement of the iron with rather small changes in their side chain conformation. In addition, the side chain of Asn177 shifted toward Asp333, although the helix α6, including Asn177, had slight movement (Figure 4). In chains B and C of Oxy: Fd^red-CAR^, the average bond lengths between mononuclear iron–Asp333OD1 and mononuclear iron–Asp333OD2 changed from 2.0 Å to 1.9 Å and from 2.6 Å to 2.2 Å, respectively, which made the coordination of the carboxylate residue to nonheme iron clearly bidentate (Table 3). Similar conformational changes were reported in nitrobenzene 2,3-dioxygenase (NBDO) bound with substrates [20]. In many iron enzymes that utilize dioxygen, the long helices containing some ligands were to some extent distorted [41,42]. Because such distortion has been considered to provide a dioxygen accommodation pathway or increase conformational flexibility, it was also suggested for CARDO that the distortion in the α11 helix may be involved in creating an accommodation route for the dioxygen molecule. In the reduced structure of OMO-O without the substrate, it was also reported that reduction of the Rieske cluster displaced the nonheme iron 0.8 Å away from the substrate-binding site [14]. However, an electron nuclear double resonance (ENDOR) study on naphthalene-bound NDO-O with a reduced Rieske cluster showed that the substrate was displaced 0.5 Å away from the nonheme iron compared with that in an oxidized Rieske cluster [27,28]. In the case of CARDO-O, reduction upon substrate binding triggered displacement of the nonheme iron and residues including Asp333 away from the substrate, which increased the accessible surface at the mononuclear iron, opened room for dioxygen binding, and allowed binding of an exogenous sixth water ligand. In other words, movement of the nonheme iron away from the substrate when the Rieske cluster is reduced might act as a physical mechanism of gating oxygen reactivity. Furthermore, the conformational changes observed upon CAR-binding and reduction experimentally demonstrate the hypothesis in the previous spectroscopic studies by Wolfe et al.: dioxygen binding at the mononuclear iron site is controlled by substrate binding to the enzyme when the Rieske cluster is reduced [25,26].

**Table 3:** Average bond lengths (Å) between nonheme iron and atoms in the catalytic cycle

**State**^***a***^	**H183**	**H187**	**D333**	**D333**	**CAR**	**CAR**	**Peroxide**	**Peroxide**
	**NE2**	**NE2**	**OD1**	**OD2**	**C1**	**C9a**	**O1**	**O2**
(1) Resting	2.1	2.2	2.1	2.8	–	–	–	–
(Oxy: Fd^rest^)								
(2) Reduced	2.1	2.1	2.0	2.6	–	–	–	–
(Oxy: Fd^red^)								
(2’) CAR-bound	2.1	2.3	2.0	2.6	4.3	4.2	–	–
(Oxy: Fd^CAR^)								
(3) Reduced CAR-bound	2.2	2.1	A^*b*^:1.9	A^*b*^:2.7	4.6	4.7	–	–
(Oxy: Fd^red-CAR^)			B&C^*b*^:2.0	B&C^*b*^:2.2				
(3’) Oxygen-bound	2.1	2.1	2.0	2.7	–	–	2.6	2.0
(Oxy: Fd^O2^)								
(4) CAR- and oxygen-bound	2.1	2.1	1.9	2.6	4.6	4.5	A^*b*^:2.5	A^*b*^:1.9
(Oxy: Fd^CAR-O2^)							B&C^*b*^:1.8	B&C^*b*^:1.8

^*a*^ Numbers correspond to Figure 1.

^*b*^ Alphabet indicates the chain name.

### The oxygen-bound complex structure [oxy: Fd^O2^, (3’)]

To obtain the oxygen-bound complex structure, Oxy: Fd^red^ crystals were transferred to a crystallization solution for oxygen exposure. As a result, clear electron density corresponding to two atoms, such as oxygen atoms, was observed in all three chains (Additional file 1: Figure S1B and Table 2). As shown in Figure 2, Rieske cluster of the Oxy: Fd^O2^ crystal was proven to be oxidized. The positions of these exogenous atoms were roughly similar to those of dioxygen bound to NDO-O [13]. Although the chemical nature of these exogenous atoms was not clear, we tentatively added a dioxygen molecule to the electron density in the model by referring to NDO-O [13]. To determine the type of dioxygen species present in the crystals, we refined the dioxygen species with an unrestrained O–O distance. As a result, the O–O distance was refined to be approximately 1.5-1.6 Å. Such a distance between oxygen atoms suggests that the dioxygen species was a peroxide species. Thus, the O–O distance of the putative peroxide molecule was refined under the restraint of 1.45 Å, and we determined the oxygen-bound complex structure [Oxy: Fd^O2^, (3’) in Figure 1.

In the final model, the O–O distance in the putative peroxide molecule was refined to 1.45 Å, and the average temperature factors of the oxygen atoms were refined to 37.2 Å^2^ (O1) and 35.6 Å^2^ (O2), which were similar to those of all residues (29.7 Å^2^) and the nonheme iron (31.0 Å^2^) in chains A to C. The O2 atom was liganded to the nonheme iron (2.0 Å), whereas the O1 atom was remote from the nonheme iron (2.6 Å) (Table 3). In the binary complex structure of NDO-O with dioxygen, the distances of two oxygen atoms from the iron were comparable (2.1 and 2.3 Å) [13]. Thus, the orientation of dioxygen bound to the nonheme iron between the active sites of CARDO-O and NDO-O was significantly different, and the dioxygen molecule was found to bind with the Oxy: Fd^O2^ crystal in an end-on fashion (Additional file 1: Figure S1B and 3D). Previously, the mononuclear iron(III) species with end-on and side-on peroxides was proposed or identified in the catalytic cycles of the antitumor drug bleomycin and various enzymes [43]. Roelfes et al. reported the characterization of the model complex of Fe(III)-peroxo species with resonance Raman spectroscopy and suggested that the Fe(III)-peroxo complex (side-on binding) and Fe(III)-hydroperoxo complex (end-on binding) were interchangeable by treatment with an appropriate acid or base [41]. In the case of the Oxy: Fd^O2^ structure, because the pH of the crystallization solution was low (around 5.5), the iron-peroxo species in Oxy: Fd^O2^ was proposed to be Fe(III)-hydroperoxo. Studies of NDO and BZDO using nitric oxide and dioxygen, however, have shown that reactivity at the active site is regulated by both the redox state of the Rieske cluster and the presence of substrate. Especially with dioxygen, no reaction occurs in the absence of an aromatic substrate if the Rieske cluster is oxidized [25,26]. Thus, it was expected that substrate binding regulates the manner of dioxygen binding in the CARDO Oxy component in either a side-on or end-on binding fashion (see next section). Furthermore, despite dioxygen bound to the active site, the distances between nonheme iron and oxygen atoms of the carboxyl group of Asp333 in Oxy: Fd^O2^ were almost identical to those of Oxy: Fd^red^ (2.0 Å and 2.7 Å vs. 2.0 Å and 2.6 Å, respectively; Table 3), and no changes were observed in the positions of the nonheme iron between the two structures.

### The CAR- and oxygen-bound complex structure [oxy: Fd^CAR-O2^, (4)]

The structure obtained from air-treated Oxy: Fd^red-CAR^ crystals during CAR-soaking seemed to bind with both CAR and dioxygen. As shown in Additional file 1: Figure S1C, the *F*_o_-*F*_c_ map obtained during the course of crystallographic refinement of this structure showed a large residual density beside the nonheme iron, suggesting the existence of a dioxygen species. We refined the dioxygen species as described in the subsection “The Oxygen-Bound Complex Structure”, and the O–O distance was refined to approximately 1.5 Å. Therefore, the dioxygen species was likely a peroxide species.

In the final model, the distance between two atoms in the putative peroxide molecule was refined to 1.44 Å [Oxy: Fd^CAR-O2^; (4) in Figure 1. Considering the fact that Rieske clusters of Oxy: Fd^O2^ were experimentally proven to be oxidized, Rieske clusters of Oxy: Fd^CAR-O2^ crystals were proposed to be oxidized. Based on Fe–O bond lengths, the complex structure was likely to be Fe(III)-(hydro)peroxo species that were found in NDO-O bound with both indole and oxygen and the BZDO-O peroxide shunt reaction [13,26]. Moreover, the electron densities corresponding to dioxygen species above the nonheme iron in the *F*_o_*F*_c_ map differed in all chains of Oxy: Fd^CAR-O2^. In chains B and C, the locations of dioxygen species were similar to those of the dioxygen-bound NDO-O with indole (side-on fashion) [13], which indicates that the average bond lengths between mononuclear iron–Asp333OD1 and –Asp333OD2 were both approximately 1.8 Å and bound in a side-on fashion (Figure 3E and Table 3). The electron density of the dioxygen molecule at the active site of chain A was identical to that of Oxy: Fd^O2^ (the bond lengths between mononuclear iron and oxygen atoms of Asp333 were 2.6 Å and 2.0 Å, respectively, in an end-on fashion) (Tables 2 and 3). In addition, the electron density of CAR was not found in the active site of chain A (Table 2). The average distances between peroxide O1 atom–CAR C9a atom and peroxide O2 atom–CAR C1 atom were both approximately 2.9 Å. This binding manner agrees with the fact that CARDO-O catalyzes the angular dioxygenation for CAR.

In chains B and C, the average temperature factors of the oxygen atoms were refined to 53.6 Å^2^ (O1) and 54.9 Å^2^ (O2), respectively, and the values were higher than those of all residues (40.3 Å^2^) and the nonheme iron (39.9 Å^2^). The bound CAR also had temperature factors comparable with those of the dioxygen species (48.2 Å^2^) in those chains. These results indicate that dioxygen species and CAR had high but not full occupancy. Further assessment of Oxy: Fd^CAR-O2^ was carried out using a simulated annealing omit map for each ligand atom. The omit map for each atom of the diatomic molecule resulted in a residual electron density at the position of the omitted atom (Additional file 1: Figure S1C). Considering the fact that CARDO showed scarce activity at the low pH employed in crystallization (Nam et al., unpublished results), Oxy: Fd^CAR-O2^ in chains B and C might be close to the structure of a reactive complex [(5) in Figure 1].

**Figure 5:**
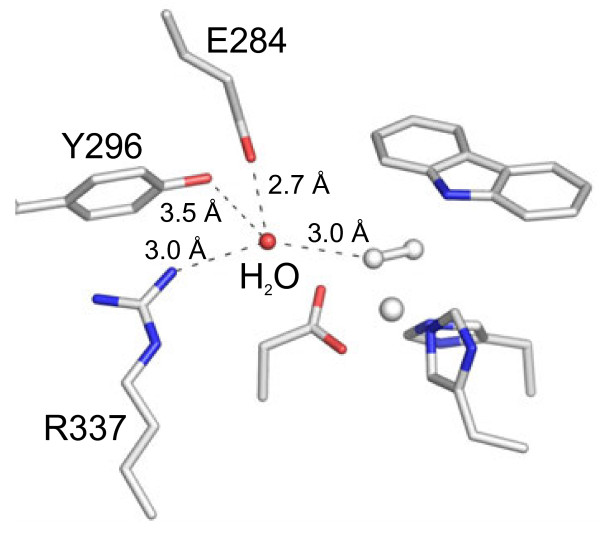
**Water molecule proposed as a proton donor for the peroxide.** The water molecule had hydrogen-bonds to Glu284, Tyr296, and Arg337, which were conserved perfectly in all reported CARDO-Os.

Comparison of the dioxygen species in two dioxygen-binding structures, (3’) and (4) in Figure 1, indicated that the orientation of dioxygen bound to the nonheme iron was roughly similar but that the binding manner was not (side-on in chains B and C of Oxy: Fd^CAR-O2^ and end-on in all chains of Oxy: Fd^O2^) (Figure 3F and Table 3). Based on our data and the proposal by Wolfe et al. [25,26], we speculated that the above-mentioned difference in the dioxygen-binding manner was due to whether the substrate was bound to the active site. That is, substrate binding might ensure that reactive oxygen species are prompted to bind to the desirable position (fashion). In addition, from superimposition, CAR was found in almost the same position as that found in Oxy: Fd^red-CAR^ (3) and Oxy: Fd^CAR^ (2’) (Figure 3G). The position of the dioxygen molecule was similar to that found in the indole- and oxygen-bound NDO-O [13], suggesting that this position is a common place for binding of activated dioxygen species in RO-Os (Figure 3E). The distances between the nonheme iron and oxygen atoms (OD1 and OD2) of Asp333 in Oxy: Fd^CAR-O2^ were similar to those in Oxy: Fd^CAR^ (2’) and Oxy: Fd^O2^ (3’) (Table 3), and the nonheme iron moved to a position close to those in Oxy: Fd^CAR^ and Oxy: Fd^O2^ (Figure 3H). The movement resulted in the fix of reactive oxygen species at a suitable place for binding in Oxy: Fd^CAR-O2^, thereby seemingly catching oxygen atoms with substrate strongly to minimize the risk of leakage and unexpected reaction. Although further investigation would be needed to determine what Fe(III)-peroxo species is protonated, protons are probably delivered through a water molecule connected to Glu284, Tyr296, and Arg337, which formed the substrate-binding pocket, because these three amino acid residues are conserved perfectly in all reported CARDO-Os (Figure 5).

## Conclusions

The series of Oxy:Fd complex structures represented sequential steps along with the single turnover reaction, although the structure bound with product has not been obtained until now. Our findings in the three structures in this study have provided several important implications for conformational changes in the catalytic cycle of ROs. In Oxy: Fd^red-CAR^, substrate binding under the reduced condition triggers a shift of both nonheme iron and its ligand residues, allowing dioxygen to bind to the active site. The Oxy: Fd^CAR-O2^ structure showed dioxygen bound to the nonheme iron and it was likely to be an Fe(III)-(hydro)peroxo species. Structural comparison of Oxy: Fd^O2^ and Oxy: Fd^CAR-O2^ demonstrated the possibility that regulation by the substrate ensures that reactive oxygen species bind to the desirable position in an appropriate side-on fashion.

The scheme of the catalytic mechanism in ROs by integration of reported findings in various structural and kinetic studies, including ours, was proposed as follows: i) The reduced ferrous iron in the active site and the oxidized Rieske cluster is the first state in the catalytic cycle [(1) in Figure 1. ii) When the Rieske cluster is reduced by receiving one electron from redox partner, the substrate binds to the active site. As a result, several conformational changes (e.g., movements of the nonheme iron and the ligand residue) occur, creating space for oxygen binding [(2) and (3)]. iii) Dioxygen bound in a side-on fashion to the active site of nonheme iron is activated by reduction to the peroxo state [Fe(III)-(hydro)peroxo] [(4)]. This state may react directly with the bound substrate, or O–O bond cleavage may occur to generate Fe(V)-oxo-hydroxo species prior to the reaction. iv) After producing a *cis*-dihydrodiol, the product is released by reduction of the nonheme iron with the second electron from the redox partner as suggested by Wolfe et al. [25,26], and then the state returns to the starting state [(5) to (1)].

Some previous reports have suggested that oxygen activation at RO-O nonheme iron sites occur through Fe(IV) and Fe(V) states [25,44,45]. However, Ferraro et al. proposed that no higher-order oxidation states need to be invoked for a concerted mechanism leading to *cis*-dihydroxylation reactions catalyzed by ROs [10]. This hypothesis involves the formation of an Fe(III)-(hydro)peroxo complex, which is supported by several reports [13,19,33,46,47]. Thus, whether the O–O bond must break before reaction with aromatic substrates remains unclear. A recent mechanistic study of NDO using diagnostic probe molecules, however, suggested that their monooxygenation reaction is mediated by an Fe(V)-oxo-hydroxo intermediate [48]. Alternatively, this high oxidation state would be difficult to achieve in a nonheme ligand environment [49]. Further study is required to elucidate the nature and reactivity of the iron-dioxygen species in ROs. The results presented in this paper provide an additional basis for further structural investigations of oxygen binding and activation in biological systems.

In addition, it is of great interest when and how the redox partner is associated and then disassociated to the oxygenase component in the catalytic cycle because few structure-based interpretations of the interactions among RO components have been reported. Therefore, the determination of complex structures between oxygenase and redox partner by X-ray crystallography under redox-state-controlled conditions will be important.

## Methods

### Purification and crystallization

CARDO-O from *Janthinobacterium* sp. J3 and CARDO-F from *Pseudomonas resinovorans* CA10 were used as CARDO components. Both components were purified as described previously [15,38]. CARDO-F from CA10 has one amino acid mismatch with that from J3, showing 99.1% identity at amino acid sequence level. The electron transferability between the purified CARDO-O of J3 and CARDO-F of CA10 was confirmed by detecting oxygenase activity for CAR, and differences of the catalytic properties between heterologous and natural combinations were hardly detected for CAR [15,17,50]. The crystallization condition for Oxy:Fd was the same as that described in our previous study [49].

### Preparation of oxy: Fd^red-CAR^ crystals

After Oxy: Fd^rest^ crystals were soaked for 20 min [50] in a crystallization solution containing 20% (v/v) glycerol, 5% (v/v) dimethylsulfoxide, 0.25% (w/v) CAR, and 20 mM sodium dithionite within a well of a microbatch plate (Hampton Research, Aliso Viejo, CA, USA) and covered with Al's oil (Hampton Research) at 20°C, the crystals were flash-frozen in a nitrogen stream at 100 K.

### Preparation of oxy: Fd^O2^ crystals

Crystals of Oxy: Fd^rest^ were soaked in a crystallization solution containing 20% (v/v) glycerol and 20 mM sodium dithionite covered with Al's oil for 20 min at 20°C. The crystals were then transferred to a crystallization solution containing glycerol without sodium dithionite at 20°C under aerobic condition. Finally, the crystals were cryo-cooled in liquid nitrogen.

### Preparation of oxy: Fd^CAR-O2^ crystals

The crystals of Oxy: Fd^red-CAR^, which were prepared as described above, were transferred to a crystallization solution containing 20% (v/v) glycerol, 5% (v/v) dimethylsulfoxide, and 0.25% (w/v) CAR without sodium dithionite for 10 min at 20°C under aerobic condition. The crystals were then cryo-cooled in liquid nitrogen.

### Measurement of UV-visible absorption spectra of the rieske cluster

Absorption spectra from the mixed solution of CARDO-O and CARDO-F, which was used for crystallization (2 mg/ml in 50 mM Tris–HCl,pH 7.5), were taken at room temperature as described previously [51]. Absorption spectra of crystals of Oxy: Fd^rest^, Oxy: Fd^red^, and Oxy: Fd^O2^ were measured with a microspectrophotometer under cryo-stream of nitrogen at 100 K [16,39].

### Data collection

The X-ray diffraction data for the three complex crystals were collected at 100 K on the beamline AR-NW12A at Photon Factory and BL41XU at SPring-8. All diffraction data were gathered with a wavelength of 1.0 Å and processed with HKL2000 software [52]. The data collection and processing statistics are given in Table 1.

### Molecular replacement, model building, and refinement

The structures of Oxy: Fd^rest^ (2DE5) and Oxy: Fd^CAR^ (2DE7) were used as molecular replacement models for the program Molrep [53]. Refinement and model building in the electron density map were carried out with the Quanta (Accelrys, San Diego, CA, USA) and Xtalview [54] programs. Refinement was carried out using the Refmac5 program in CCP4 [55] and CNS 1.1 [56] by gradually adding water molecules. The stereochemistry of the model was analyzed using the Procheck [57], Whatcheck [58], and RAMPAGE [40] programs. All descriptions of crystal structures were generated using PyMOL [59]. Modeling of the exogenous atoms in oxygen-bound complex structures was carried out following the procedure described by Karlsson et al. [13]. In brief, we first refined those crystal structures without exogenous atoms. The resultant 2*F*_o_*F*_c_ and *F*_o_*F*_c_ maps showed a large electron density beside the nonheme iron. One oxygen atom was then modeled in the electron density. The crystallographic refinement with one oxygen atom, however, still gave an excess residual electron density besides the oxygen atom. Therefore, we placed a dioxygen molecule into the electron density of the map and refined the structure. The obtained *F*_o_*F*_c_ map for the structure showed no residual electron density around the nonheme iron, but the chemical nature of these exogenous atoms was unclear. To determine the type of dioxygen species present in the crystals, we refined the dioxygen species with an unrestrained O–O distance. Further assessment for this model was carried out using a simulated annealing omit map for each ligand atom. The omit map for each atom of the diatomic molecule resulted in a residual electron density at the position of the omitted atom.

### Superimposition of the protein structures

Least-squares comparisons of various complex structures were carried out using the programs Coot [60] and PyMOL [59].

### Accession numbers

The atomic coordinates and structure factors have been deposited in the Protein Data Bank, http://www.pdb.org (PDB codes 3VMG, 3VMH, and 3VMI for reduced carbazole-bound complex, oxygen-bound complex, and carbazole- and oxygen-bound complex, respectively).

## Abbreviations

BZDO, Benzoate 1,2-dixoygenase; BZDO-O, Oxygenase component of BZDO; CAR, Carbazole; CARDO, Carbazole 1,9a-dioxygenase; CARDO-F, Ferredoxin component of CARDO; CARDO-O, Oxygenase component of CARDO; CARDO-R, Ferredoxin reductase component of CARDO; ENDOR, Electron nuclear double resonance; Fd, CARDO-F of Pseudomonas resinovorans CA10; NBDO, Nitrobenzene 2,3-dioxygenase; NDO, Naphthalene 1,2-dioxygenase; NDO-O, Oxygenase component of NDO; OMO, 2-oxoquinoline 8-monooxygenase; OMO-O, Oxygenase component of OMO; Oxy, CARDO-O of Janthinobacterium sp. J3; PDB, Protein Data Bank; PDO, Phthalate 4,5-dioxygenase; PDO-O, Oxygenase component of PDO; RMSD, Root mean square deviation; RO, Rieske nonheme iron oxygenase system; RO-O, Oxygenase component of RO.

## Competing interests

The authors declare that they have no competing interests.

## Authors’ contributions

HN and YA designed this research. YA performed all experiments in this study. ZF aided model building and refinement. YU, KI, and HY supervised this work. The manuscript was written and figures and tables were prepared by YA and HN. All authors read and approved the final manuscript.

## Supplementary Material

Additional file 1: **Title of data: Figure S1 Illustrations of the active site in complex structures.** Description of data: All panels show the active site of chain C in complex structures. (A) Binding of CAR at the active site of Oxy: Fd^red-CAR^ [(3) in Figure 1]. The omitted density map (*F*_o_-*F*_c_) is shown as a black wire mesh and contoured at 3.5 δ. Only the nonheme iron (sphere), waters liganded to the iron (small sphere), iron-coordinated residues (H183, H187, and D333; stick), and CAR (stick) are shown and colored magenta. (B) Binding of dioxygen to the nonheme iron in Oxy: Fd^O2^ [(3’) in Figure 1]. Dioxygen molecule is shown as stick-and-ball and the presentation of other atoms is identical to that in panel (A), and their color coding is salmon. The black map is an *F*_o_-*F*_c_ omit map (5 δ) of both dioxygen atoms. The green and red maps are *F*_o_-*F*_c_ omit maps (5 δ) for the O1 atom and O2 atom, respectively, of the dioxygen molecule. (C) Binding of dioxygen molecule at the active site with the presence of CAR in Oxy: Fd^CAR-O2^ [(4) in Figure 1]. Color coding is white. The *F*_o_-*F*_c_ omit maps (2.5 δ) of CAR, both dioxygen atoms, the O1 atom, and the O2 atom are colored cyan, black, green, and red, respectively. Click here for file
