# Supplementary figures and images for: Structural insight into the substrate- and dioxygen-binding manner in the catalytic cycle of rieske nonheme iron oxygenase system, carbazole 1,9a-dioxygenase
Source: BMC Struct Biol. 2012 Jun 24;12:15. doi: 10.1186/1472-6807-12-15 (PMC3423008; doi:10.1186/1472-6807-12-15)

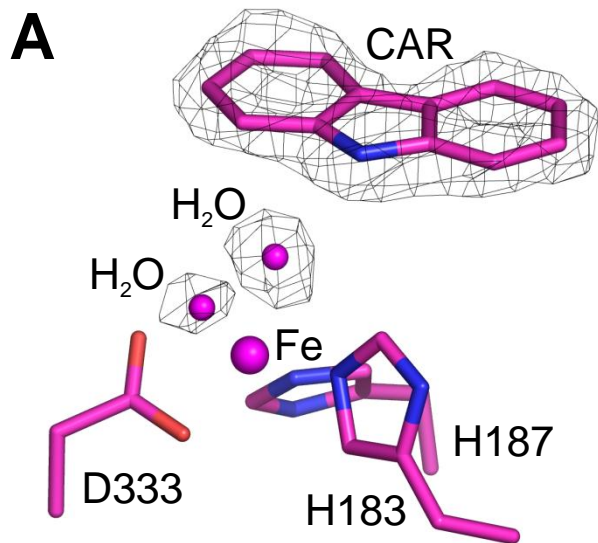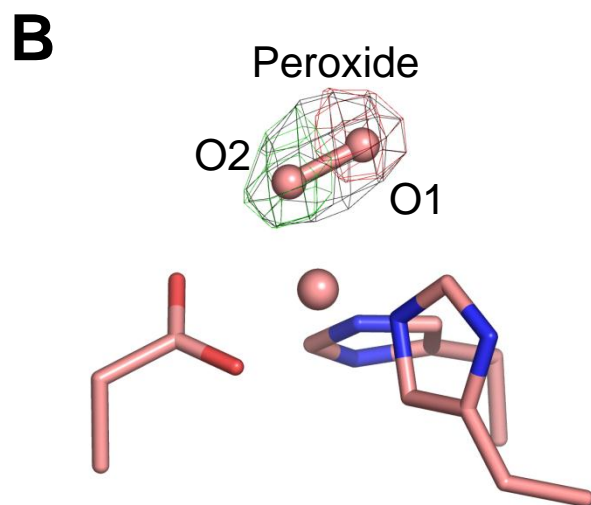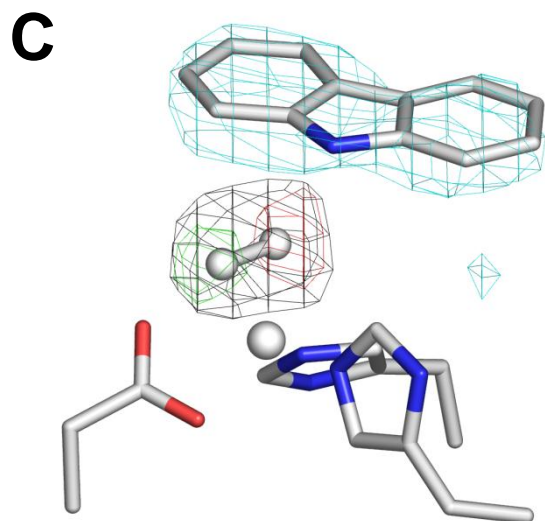

Supplement: Additional file 1: — Title of data: Figure S1 Illustrations of the active site in complex structures. Description of data: All panels show the active site of chain C in complex structures. (A) Binding of CAR at the active site of Oxy: Fdred-CAR [(3) in Figure 1]. The omitted density map (Fo-Fc) is shown as a black wire mesh and contoured at 3.5 δ. Only the nonheme iron (sphere), waters liganded to the iron (small sphere), iron-coordinated residues (H183, H187, and D333; stick), and CAR (stick) are shown and colored magenta. (B) Binding of dioxygen to the nonheme iron in Oxy: FdO2 [(3’) in Figure 1]. Dioxygen molecule is shown as stick-and-ball and the presentation of other atoms is identical to that in panel (A), and their color coding is salmon. The black map is an Fo-Fc omit map (5 δ) of both dioxygen atoms. The green and red maps are Fo-Fc omit maps (5 δ) for the O1 atom and O2 atom, respectively, of the dioxygen molecule. (C) Binding of dioxygen molecule at the active site with the presence of CAR in Oxy: FdCAR-O2 [(4) in Figure 1]. Color coding is white. The Fo-Fc omit maps (2.5 δ) of CAR, both dioxygen atoms, the O1 atom, and the O2 atom are colored cyan, black, green, and red, respectively. [file 1472-6807-12-15-S1.pdf]
